# Obesity, Inflammation, Growth, and Metabolism: Evolution of Understanding and Evolving Functions of Old and New Peptides

**DOI:** 10.3390/jcm12123913

**Published:** 2023-06-08

**Authors:** Maria Elisabeth Street

**Affiliations:** 1 Department of Medicine and Surgery, University of Parma, 43125 Parma, Italy; mariaelisabeth.street@unipr.it; 2 Unit of Paediatrics, University Hospital of Parma, 43126 Parma, Italy

Obesity is a well-known low-grade chronic inflammatory disease that leads to metabolic derangements, cardiovascular complications, changes in growth, timing of puberty, bone formation, and changes in the ability to face infection. Furthermore, obesity has an increased risk of cancer. Since childhood, circulating adipocytokines and proinflammatory cytokines are reported to be increased in the circulation, and have proven relationships with the IGF system, are related with insulin sensitivity, longitudinal growth and glucose metabolism. These hold in conditions of severe genetic obesity such as Prader–Willi syndrome (PWS), where organic causes aggravate the psychosocial problems also present in obesity. Among these, stigma and stress have gained particular consideration recently and have highlighted the need and usefulness in clinical practice of scores such as EPICES to evaluate the deprivation and inequalities of health in healthcare centers, and the Edmonton score [1,2,3] to address clinical interventions. The remaining challenge concerns the methods of clinically and/or biochemically identifying children who are most at risk of developing complications. From this point of view, increasing data collection confirms that the “metabolic phenotype” is driven mainly by insulin resistance, fat distribution and dyslipidemia more than by body weight per se [4,5]. Comprehension of the underlying mechanisms will further help prevention and treatment, as is the case for the recent description of increased chitotriosidase activity, along with many other markers [6,7,8]. Interestingly, chitinases can have many targets, including some relevant to host infection offering an interesting link for the increased mortality rate of obese subjects during severe infection.

Inflammation is a modifier of key mechanisms regulating longitudinal growth. This has been previously shown in cystic fibrosis [9] and in other conditions characterized by chronic inflammation [10]. Moreover, this has also been demonstrated in otherwise healthy obese children, where increased IGF-II, decreased IGFBP-1, and IGFBP-2 concentrations were described along with reduced IGF-I bioactivity [11]. Reduced IGF-I bioactivity has been recently confirmed in PWS, thus suggesting that free IGF-I would be the best bio-marker to monitor growth hormone (GH) function and treatment rather than total IGF-I, and often would avoid worries and incorrect reduction of treatment regimens with GH by clinicians [12]. Regarding cystic fibrosis, insulin secretion and insulin sensitivity have shown an important interplay mostly mediated by the defects in the cystic fibrosis transductance regulator (CFTR) and characterized by both insulin resistance and reduced insulin secretion leading over the years to diabetes [13]. Since the introduction of CFTR modulators in clinical practice, whereas inflammation is reduced, effects on glucose metabolism, mediated by the above-mentioned mechanisms, are still unsatisfactory [14], although glycemic control has greatly improved from advanced technologies that are currently changing the paradigms of treatment for diabetes [15]. This prompts further research in this field.

Finally, the amount of knowledge building up in the field of obesity, inflammation, metabolism, and growth is such that machine learning and big data analysis should be applied, finally providing guidance for future research directions. These could take iinto account all data arising from “omic” studies and those considering the effects of environmental pollution.
